# Volumetric Infiltration of Brushite and Monetite Cements into Laser Powder Bed Fusion Ti-6Al-4V Lattices: A Feasibility Study of Lattice Geometry and Cement Formulation

**DOI:** 10.3390/ma19183973

**Published:** 2026-09-18

**Authors:** Tiffany Kisway, Ella Lawrence, Melissa Trask, Lauren MacRae, Meredith McCorkell, Emily Parsons, Farmehr Asefzadeh, David T. Wu, Pierre-Luc Michaud, Donald Paul Bishop, Zeeshan Sheikh

**Affiliations:** 1 Biomaterials & Applied Oral Sciences (BAOS), Faculty of Dentistry, Dalhousie University, Halifax, NS B3H 4R2, Canada; tiffany.kisway@dal.ca (T.K.); el968204@dal.ca (E.L.); e.parsons@dal.ca (E.P.); 2 Dental Clinical Sciences (DCS), Faculty of Dentistry, Dalhousie University, Halifax, NS B3H 4R2, Canada; fr634704@dal.ca (F.A.); pl.michaud@dal.ca (P.-L.M.); 3 Faculty of Science, Dalhousie University, Halifax, NS B3H 3Z1, Canada; lr387297@dal.ca; 4 Department of Mechanical Engineering, Dalhousie University, Halifax, NS B3J 1B6, Canadapaul.bishop@dal.ca (D.P.B.); 5 Faculty of Engineering and Health Sciences, Biomedical and Materials Engineering, McMaster University, Hamilton, ON L8S 4L7, Canada; 6 School of Biomedical Engineering (SBME), Faculty of Medicine, Dalhousie University, Halifax, NS B3H 4R2, Canada; 7 Department of Oral Medicine, Infection, and Immunity, Harvard School of Dental Medicine, Boston, MA 02115, USA; davidt_wu@hsdm.harvard.edu; 8 Faculty of Dental Medicine and Oral Health Sciences, McGill University, Montreal, QC H3A 1G1, Canada; 9 Faculty of Dentistry, University of Toronto, Toronto, ON M5G 1G6, Canada

**Keywords:** 3D-printing, titanium, calcium phosphates, brushite, monetite, coatings, laser powder bed fusion, craniofacial, feasibility study, micro-CT

## Abstract

Craniofacial defects following maxillectomy present major reconstructive challenges, and although autologous vascularized free flaps remain standard, they carry donor-site morbidity and surgical burden. Additive manufacturing and bioactive coatings enable hybrid metallic–ceramic constructs; however, whether a bioactive phase can be delivered throughout the internal volume of an architected metal lattice, rather than only onto its surface, remains poorly characterized. In this feasibility study, laser powder bed fusion (LPBF) was used to fabricate porous Ti-6Al-4V lattices (5 mm diameter) in two geometries (octagon and grid) at nominal strut spacings of 500 and 1000 µm. Resorbable dicalcium phosphate cements of β-tricalcium phosphate and monocalcium phosphate monohydrate were infiltrated at two powder-to-liquid ratios (P/L 0.5 and 0.75) to form brushite, then thermally dehydrated to monetite. X-ray diffraction confirmed phase purity and brushite-to-monetite conversion. Micro-computed tomography showed infiltration to the lattice core, with calcium phosphates across both geometries and pore sizes; both workable formulations were compatible with the infiltration procedure. These results establish preliminary processing conditions for volumetric biofunctionalization of porous titanium and provide a defined basis for subsequent geometric, mechanical, adhesion, and biological evaluation.

## 1. Introduction

Additive manufacturing has emerged as a transformative approach in the development of functional biomaterials for medical applications, enabling unprecedented control over implant geometry, internal architecture, and material distribution [1]. Three-dimensional (3D) printing technologies allow fabrication of porous, load-bearing structures with complex geometries and patient-specific designs that are difficult to achieve using conventional subtractive manufacturing approaches [2,3,4,5]. Among these technologies, laser powder bed fusion (LPBF) enables the precise fabrication of metallic scaffolds with controlled lattice architecture, surface roughness, and porosity, making it particularly attractive for biomedical implant development [6].

Large craniofacial and maxillofacial bone defects, including those resulting from oncologic resection, trauma, or congenital deformities, present substantial reconstructive challenges due to the complex anatomy and functional demands of the craniofacial skeleton [7,8]. Current gold-standard reconstruction approaches rely primarily on autologous bone grafts or vascularized free flaps, which are associated with donor-site morbidity, limited donor site availability, prolonged operative times, and variable long-term outcomes [9,10]. From a clinical perspective, the maxilla presents a uniquely challenging environment for implant-based reconstruction. Compared with the mandible, maxillary bone typically exhibits lower density, thinner cortical plates, and a predominance of trabecular architecture, often compounded by post-extraction resorption and proximity to the maxillary sinus. These anatomical and biological constraints emphasize the need for implants that not only provide mechanical stability but also promote rapid and homogeneous bone formation throughout the scaffold structure. Consequently, there is increasing interest in alternative implant-based strategies capable of providing structural support while simultaneously facilitating reliable bone regeneration.

Titanium alloys such as Ti-6Al-4V (Ti64) exhibit excellent mechanical strength, corrosion resistance, and biocompatibility, making them widely used for load-bearing craniofacial and dental implants [11,12,13,14]. Additively manufactured titanium lattices can introduce controlled porosity that promotes osseointegration and bone ingrowth [1]. However, smooth titanium surfaces are relatively bioinert and primarily support passive osteoconduction, providing limited biochemical cues to actively stimulate bone regeneration within scaffold interiors. Surface and compositional modifications are therefore increasingly explored to enhance the biological performance of metallic implants. Surface engineering strategies have been shown to influence protein adsorption, osteoblast activity, inflammatory responses, and bacterial adhesion, all of which affect implant integration and long-term success [15,16,17].

One promising approach involves integrating calcium phosphate (CaP) biomaterials within porous titanium scaffolds to introduce bioactivity while maintaining the mechanical strength of the metallic framework [18]. Calcium phosphate biomaterials are widely used in bone regeneration due to their osteoconductive properties and chemical similarity to the mineral phase of bone [19]. A range of CaP chemistries, including amorphous calcium phosphates, hydroxyapatite, and other crystalline phases, have been investigated for coatings and graft substitutes, with biological performance influenced by composition, crystallinity, solubility, microstructure, and surface area [20]. Among these materials, dicalcium phosphate cements (DCPs), including brushite (dicalcium phosphate dihydrate) and monetite (dicalcium phosphate anhydrous), have attracted considerable interest due to their moldability, resorbability, and ability to support new bone formation under appropriate physiological conditions [21].

The brushite–monetite system is particularly attractive for regenerative applications that benefit from early ionic release and gradual replacement by bone, and these materials have been investigated as bone graft substitutes and drug-delivery matrices [19]. Brushite cements can undergo physicochemical transformations during implantation and degradation, potentially influencing their resorption behavior and biological performance [22]. Monetite, which represents the anhydrous form of dicalcium phosphate, has been reported to exhibit different dissolution characteristics and may provide more rapid resorption under certain physiological conditions [23]. These properties make brushite and monetite promising candidates for incorporation within porous scaffolds designed to promote early cellular infiltration and bone regeneration.

Hybrid constructs that combine 3D-printed titanium lattices with CaP phases have been proposed to leverage the mechanical reliability of metallic scaffolds alongside the bioactivity of calcium phosphates [18]. In principle, CaP phases incorporated within a porous titanium architecture may enhance early osteoconduction and facilitate cellular migration into the scaffold interior. Despite the extensive literature describing porous titanium scaffolds and CaP-based coatings or graft substitutes, comparatively little attention has been given to the combined influence of lattice architecture and cement formulation on volumetric infiltration of CaP phases within additively manufactured metal lattices. Prior studies have largely focused on surface coatings or bulk scaffold mechanics rather than penetration depth, spatial uniformity, or phase stability of CaP materials within internal scaffold regions; for example, surface-limited calcium phosphate deposition on additively manufactured titanium has been characterized without assessing interior distribution [24], and CaP-loaded titanium scaffolds have been evaluated for bulk mechanical behavior without quantifying internal coating penetration [18,25]. Cement infiltration into porous metallic scaffolds is governed by a combination of structural and material parameters, including pore size, lattice geometry, strut diameter, pore interconnectivity, and rheological properties such as viscosity and setting kinetics [26]. Inadequate infiltration may result in heterogeneous bioactivity, leaving internal regions of the scaffold biologically inert and limiting regenerative potential, particularly in applications where bone formation throughout the implant volume is desired.

**Figure 1:**
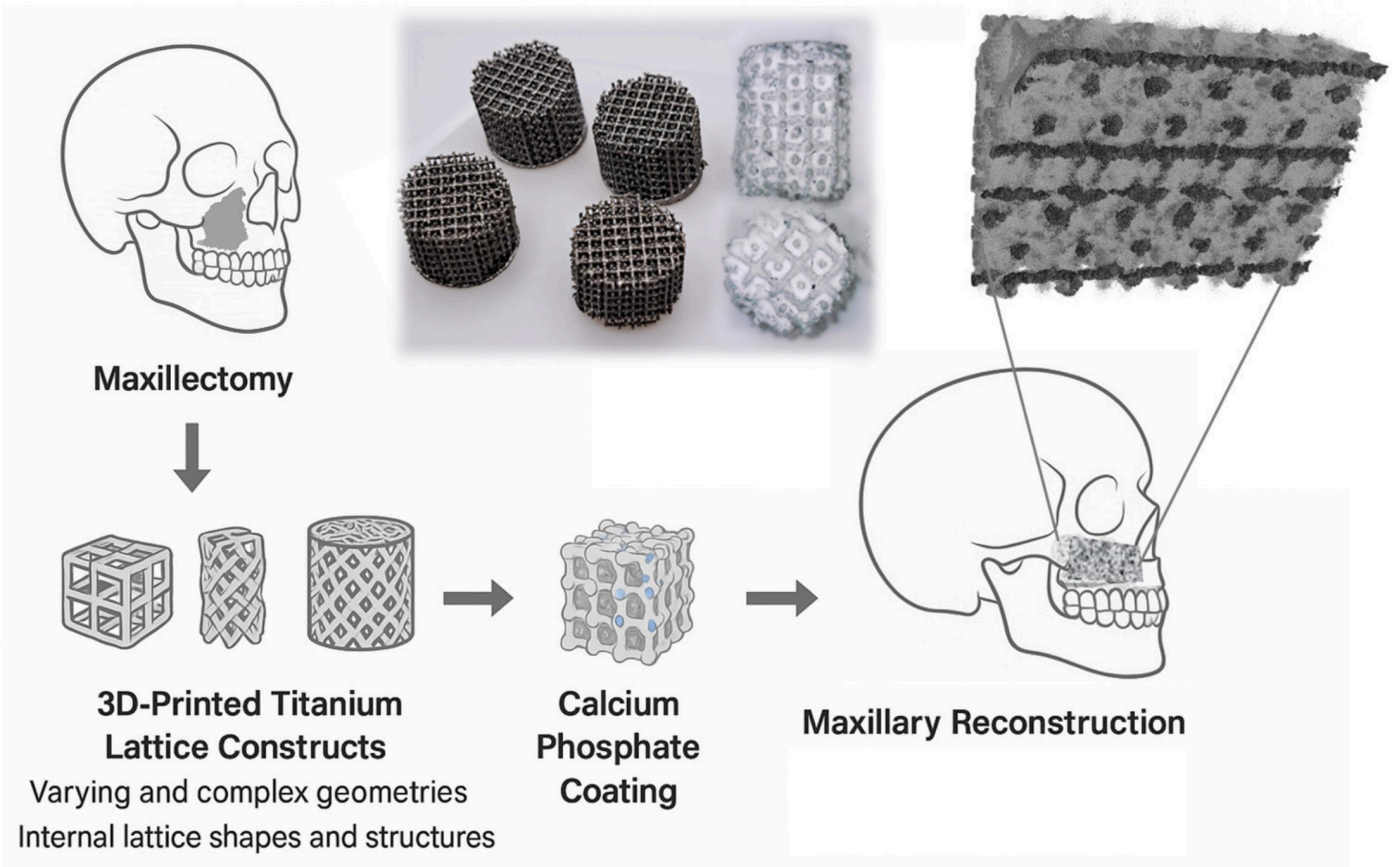
Clinical motivation and study scope. Schematic of a maxillectomy defect reconstructed with a 3D-printed titanium lattice coated with calcium phosphate and ultimately restored with an implant-supported prosthesis. The present study addresses only the calcium phosphate infiltration and phase-conversion stage indicated; mechanical, adhesion, and biological stages are the subject of subsequent work. Adapted from the figure originally appearing in the article https://www.oralhealthgroup.com/features/developing-3d-printed-titanium-constructs-with-calcium-phosphate-coatings-for-craniofacial-reconstruction-a-nserc-discovery-grant-program/ (accessed on 8 July 2026), published in the Oral Health Journal in December 2025.

The objective of this feasibility study was therefore to characterize the infiltration behavior of dicalcium phosphate cements within additively manufactured titanium lattices of varying geometry and strut spacing, and to establish preliminary processing conditions for volumetric biofunctionalization, rather than to establish statistically powered effects of these variables. Titanium lattices with grid and octagonal architectures were fabricated using LPBF with pore spacings of 500 µm and 1000 µm and a strut thickness of 150 µm. This study was deliberately limited to the infiltration and phase-formation stage of a staged development program; coating–substrate adhesion and mechanical performance were reserved for subsequent dedicated evaluation. Brushite (CaHPO_4_·2H_2_O) was prepared as a CaP cement at two powder-to-liquid ratios and introduced into the lattice structures. Brushite cements were converted to monetite (CaHPO_4_) through thermal dehydration. Surface imaging, confocal microscopy, X-ray diffraction, and micro-computed tomography (micro-CT) were used to assess spatial distribution, surface morphology, phase composition, and cement penetration. By characterizing the interplay between lattice architecture and cement formulation on internal cement penetration, this work aims to establish preliminary materials-processing conditions for volumetric biofunctionalization of architected titanium lattices. Craniofacial reconstruction is presented here as the motivating clinical context; mechanical, adhesion, and biological evaluation are defined as subsequent stages of this program and are outside the scope of the present study (Figure 1).

## 2. Materials and Methods

### 2.1. 3D Printing of Titanium Lattice Scaffolds

**Figure 2:**
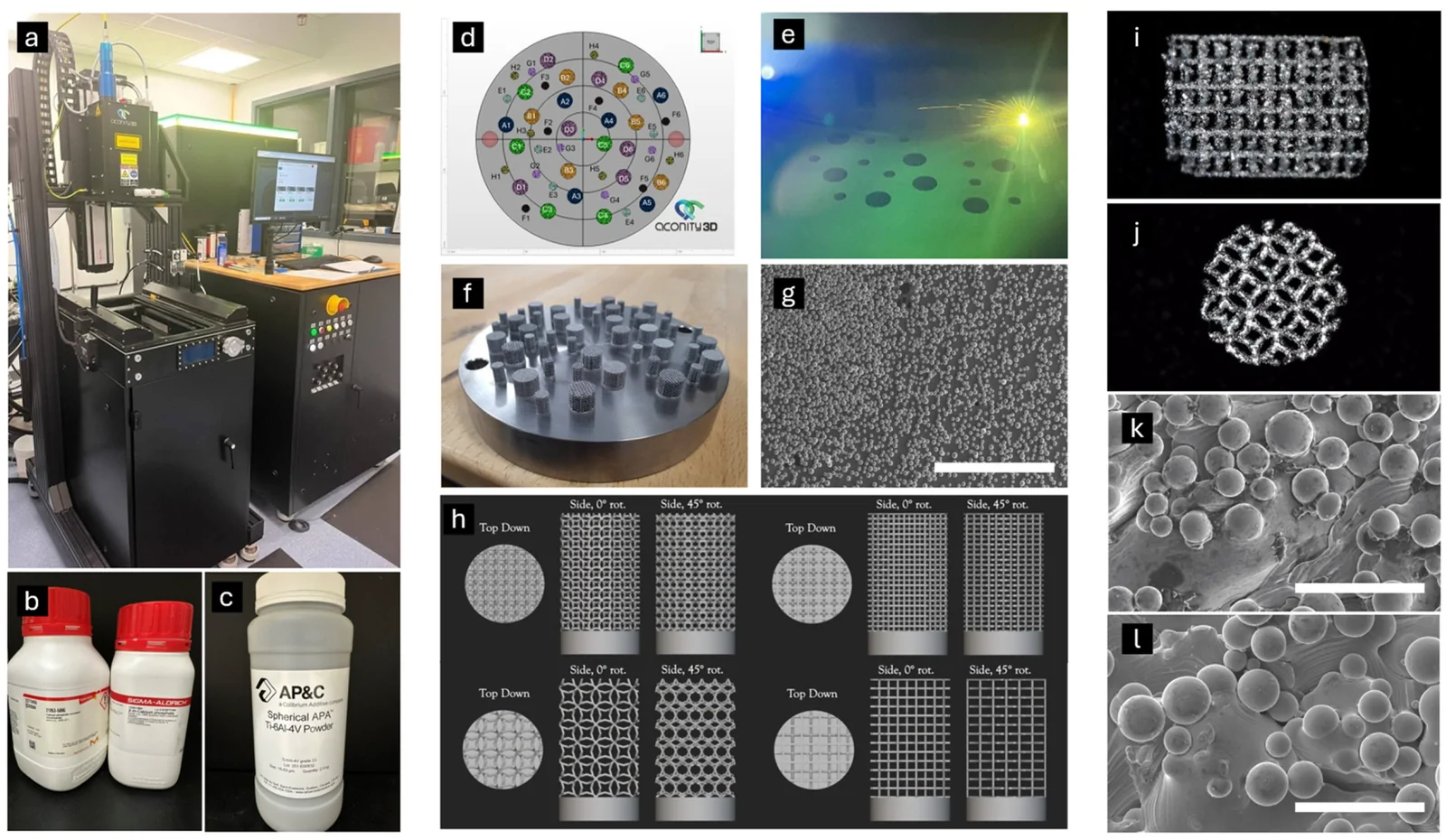
Schematic representation of materials used and fabrication processes: (**a**) Aconity LPBF machine; (**b**) β-TCP and MCPM powders; (**c**) titanium powder; (**d**) CAD build plate design; (**e**) in-progress image of LPBF printing process; (**f**) image of the build plate with fully fabricated titanium lattices after printing; (**g**) SEM image of titanium (Ti64) powder taken at 50×, scale bar represents 1000 µm; (**h**) CAD representation of lattice designs at varying angles of visualization; (**i**) digital photograph showing a side view of an octagon, 1000 µm uncoated lattice; (**j**) digital photograph showing a top view of octagon 1000 µm lattice; (**k**) SEM image of Ti64 lattice prior to ultrasonication cleaning protocol—taken at 500×, scale bar represents 100 µm; (**l**) SEM image of Ti64 lattice after undergoing ultrasonication cleaning protocol—taken at 500×, scale bar represents 100 µm; (**k**,**l**) reduction in residual Ti64 residues following sonication validates the sonication cleaning protocol.

Three-dimensional designs for titanium lattice scaffolds were initially developed using nTop 5^TM^ computational design software. Subsequently, the scaffolds were printed from plasma-atomized Ti-6Al-4V (Ti64; AP&C, Montréal, QC, Canada) powder using an Aconity Mini laser powder bed fusion (LPBF) system (GmbH, Aachen, Germany) equipped with an ytterbium (Yb)-doped fiber laser. Key operating parameters included a laser power of 250 W, scan speed of 1100 mm/s, hatch spacing of 80 µm, layer thickness of 30 µm, and an argon atmosphere (O_2_ < 50 ppm). Each scaffold was printed with a meander scan strategy on a Ti64 build plate (Figure 2f). Two nominal strut spacings (500 µm and 1000 µm) were selected and both grid and octagonal lattice architectures (Figure 2h) were produced at these spacings, resulting in four distinct lattice designs. Strut spacing was defined as the center-to-center distance between adjacent struts and therefore determines the characteristic pore dimensions of the lattice architecture. The selected spacings were chosen to represent pore sizes commonly reported to support cellular infiltration and bone ingrowth in porous biomaterial scaffolds. All lattices were fabricated with an overall diameter of 5 mm to provide a reproducible model geometry suitable for evaluating calcium phosphate cement infiltration and for potential translation to future preclinical implantation studies.

For each lattice configuration (grid 500 µm, grid 1000 µm, octagon 500 µm, and octagon 1000 µm), following fabrication, the specimens were mechanically separated from the build plate using an abrasive cut-off disc. Residual titanium from the build plate attachment points was subsequently removed using a Buehler EcoMet 30 semi-automatic grinder–polisher (ITW (Buehler), Lake Bluff, IL, USA) to obtain the final lattice specimens. Five independent specimens (*n* = 5) were prepared for each experimental condition. Calcium phosphate cement was prepared in four independently mixed batches. Brushite and monetite measurements were performed on separate specimens: monetite specimens were prepared and thermally converted independently, rather than by re-characterizing the same brushite specimens after conversion. Specimens were randomly allocated to coating conditions. Unless otherwise stated, the scanning electron microscopy, confocal microscopy, and micro-computed tomography images presented are representative images selected from the full set of specimens examined for each condition.

### 2.2. Sonication Cleaning of 3D Printed Lattices

Following mechanical removal from the build plate, post-processing ultrasonic cleaning procedures were performed to remove residual powder and fabrication-related contaminants in accordance with ASTM F2459 [27] and ISO 19227 standards [28] for cleaning additively manufactured metallic implants. Ultrasonic cleaning was conducted in a VEVOR ultrasonic bath (Vevor, New York, NY, USA) maintained at 30 °C. The cleaning protocol involved sequential sonication steps in acetone and deionized (DI) water to promote progressive removal of adhered powder particles. Specimens were first sonicated in DI water for 10 min, followed by air-drying for 10 min, and subsequently subjected to a second 5 min sonication in fresh DI water. This cycle was repeated until no macroscopically visible titanium residue was observed on the lattice surfaces. To further remove residual titanium powder from internal lattice regions, specimens were subsequently sonicated in acetone for 15 min, followed by an additional 5 min acetone sonication step, with 10 min air-drying periods between intervals. The cleaning process concluded with a final 5 min sonication in DI water and a 10 min air-drying step. Complete removal of powder and other contaminants was usually achieved in 90 min.

### 2.3. Surface Characterization of Cleaned Titanium Lattices

To validate the sonication cleaning protocol, evaluate surface morphology, and assess the surface chemistry of the titanium lattices, a suite of complementary analytical techniques was employed. Characterization was performed in accordance with risk-based material evaluation principles outlined in ISO 10993 [29], which recommend comprehensive surface and compositional analysis for implantable medical device materials to identify potential contaminants, residual manufacturing by-products, or surface irregularities that could influence biological performance.

The effectiveness of the sonication cleaning protocol was first verified using surface characterizations to confirm the absence of residual titanium particles on lattice surfaces and within internal structures (Figure 2). Light microscopy, scanning electron microscopy (SEM), and confocal laser scanning microscopy (CLSM) were used to examine lattice surfaces for residual powder and loosely adherent particulates. Energy-dispersive spectroscopy (EDS) was performed to evaluate elemental composition and confirm the absence of detectable contaminants. SEM imaging and EDS were performed using a Thermo Scientific AXIA ChemiSEM system (Thermo Fisher Scientific Inc., Waltham, MA, USA), while CLSM analysis was conducted using a Keyence VK-X1000 confocal laser scanning microscope (KEYENCE Corporation, Osaka, Japan) equipped with VK Viewer 2.5 and MultiFile Analyzer software.

Micro-computed tomography (micro-CT) was performed using a Bruker Skyscan 1272 system (Bruker, Kontich, Belgium) to generate high-resolution volumetric images of the lattice structures. Image reconstruction and analysis were conducted using NRecon (v1.67.91.46), CTVox, and CTAn (v1.15.4.0) software. Micro-CT imaging enabled visualization of internal lattice architecture and verification that no detectable residual titanium powder remained within the scaffold interior.

X-ray diffraction (XRD) phase characterization analysis was conducted using the Bruker D8 Advance (A25) diffractometer (Bruker AXSy GmbH, Karlsruhe, Germany) over a 2θ range of 20–130° with a step size of 0.02° and a scan rate of 1 s per step to confirm the crystalline phase composition of the Ti-6Al-4V alloy and verify that no secondary phases were introduced during the additive manufacturing or cleaning processes. Because the small lattice geometry was not suitable for direct XRD measurement, as the available equipment used a Bragg–Brentano setup that was not conducive to highly porous structures, an analogous Ti-6Al-4V (Ti64) specimen was fabricated using identical LPBF processing parameters and powder feedstock. This specimen underwent the same fabrication and post-processing cleaning protocol as the lattice structures and served as a representative sample for phase identification of the printed titanium material. Diffraction patterns were analyzed and graphed using Microsoft Excel.

SEM imaging conducted at magnification intervals between 25× and 1000× was used to examine the surface morphology of each uncoated titanium lattice type. Uncoated lattice types included grid and octagon geometries, printed at both 500 µm and 1000 µm strut spacings as outlined in Table 1. Together, these complementary characterization techniques enabled verification of lattice structural integrity, confirmation of Ti-6Al-4V phase composition, and validation of the cleaning protocol prior to calcium phosphate coating.

### 2.4. Coating of Titanium Lattices with Brushite and Monetite

The brushite cement precursor powder was prepared by combining β-tricalcium phosphate (β-TCP; Ca_3_(PO_4_)_2_) and monocalcium phosphate monohydrate (MCPM; Ca(H_2_PO_4_)_2_·H_2_O), which react in an aqueous environment to form dicalcium phosphate dihydrate (brushite; CaHPO_4_·2H_2_O) through an acid–base setting reaction. The reaction proceeds according to the following simplified stoichiometric relationship:

$$\beta -\mathrm{TCP}+MCPM+H_{2}O\to 2CaHPO_{4}\cdot 2H_{2}O$$

Upon addition of deionized water, dissolution of the calcium phosphate precursors occurs followed by precipitation of brushite crystals, resulting in a self-setting calcium phosphate cement matrix. The powder components were weighed at a mass ratio of 0.50 g β-TCP to 0.60 g MCPM, corresponding to the stoichiometric proportions required to favor brushite formation under aqueous conditions. Powder-to-liquid (P/L) ratios of 1.0, 0.75, and 0.5 (g/mL) were investigated to control the viscosity and penetration behavior of the cement slurry within the porous titanium lattice structures. Although three P/L ratios were initially produced, only 0.75 and 0.5 were selected for infiltration studies due to cement workability. Lower P/L ratios reduce paste viscosity and enhance capillary infiltration into porous architectures, while higher P/L ratios increase paste cohesion but limit penetration.

Titanium lattices with 500 µm and 1000 µm strut spacing, in both grid and octagonal geometries, were assigned to one of two coating formulations, prepared at a powder-to-liquid (P/L) ratio of 0.75 or 0.5. To achieve coating infiltration, individual lattice specimens were fully submerged in a 0.5 mL aliquot of cement slurry within the conical bottom of 15 mL centrifuge tubes. The tubes were then placed on a LABFISH Mini Vortex Mixer (Akmlab Scientific Zhejiang Co., Ltd., Huzhou, China) to gently agitate the suspension and promote complete penetration of the cement slurry throughout the lattice architecture. Following coating infiltration, the specimens were transferred to a vacuum oven maintained at 37 °C for 24 h to allow the brushite cement to set and dry.

Conversion of brushite (CaHPO_4_·2H_2_O) coatings to monetite (CaHPO_4_) was achieved through controlled dehydration (CaHPO_4_·2H_2_O → CaHPO_4_ + 2H_2_O) using a dry autoclave treatment (132 °C, 15 psi, 45 min). Autoclave treatment provides a controlled hydrothermal environment that facilitates dehydration of brushite while preserving the overall coating morphology. This approach has been widely used in calcium phosphate cement systems to produce monetite phases with higher resorption rates and improved dissolution behavior compared with brushite [22,23], making them particularly attractive for bone regeneration applications where controlled scaffold degradation is desired.

The complete experimental design produced sixteen unique coated lattice types, based on the combinations of lattice geometry (octagon or grid), strut spacing (500 µm or 1000 µm), P/L ratio (0.5 or 0.75), and coating phase (brushite or monetite). The workflow and experimental grouping are summarized in Table 1. All lattice specimens were weighed prior to coating, after vacuum drying for both brushite and monetite samples, and following autoclave dehydration for monetite samples to quantify coating deposition and mass changes associated with phase conversion.

### 2.5. Surface and Internal Characterization of Coated Lattices

Prior to electron microscopy analysis, coated lattice specimens were sputter-coated with a gold–palladium conductive layer to minimize surface charging. Scanning electron microscopy (SEM) and energy-dispersive spectroscopy (EDS) were performed using the Thermo Scientific AXIA ChemiSEM. SEM imaging was used to examine the surface morphology of the calcium phosphate coatings and to qualitatively assess coating coverage and thickness on the titanium struts, while EDS analysis was used to evaluate the elemental composition of the coatings and confirm the presence of calcium and phosphorus associated with the deposited calcium phosphate phases. It should be noted that EDS analysis was used for qualitative to semi-quantitative elemental assessment only, as accurate determination of Ca/P stoichiometry in thin coatings on metallic substrates is limited by interaction volume effects, surface topography, and potential signal contributions from the underlying titanium.

The crystalline phase composition of the coatings was analyzed using X-ray diffraction (XRD) with a Bruker D8 Advance (A25) diffractometer using a Bragg–Brentano focusing geometry. Diffraction scans were performed over a 2θ range of 10° to 80°, corresponding to the characteristic diffraction peaks of brushite and monetite, with a step size of 0.02° and a counting time of 1 s per step. The resulting diffraction patterns were compared with reference patterns from the Joint Committee on Powder Diffraction Standards (JCPDS) database to confirm the presence of brushite and monetite phases.

Micro-computed tomography (micro-CT) was performed using the Bruker Skyscan 1272 system to evaluate the internal distribution and penetration of the calcium phosphate coatings within the lattice structures.

Micro-computed tomography (micro-CT) was performed using a Bruker SkyScan 1272 system. Scans were acquired at an isotropic voxel size of 2.0 µm, source voltage of 100 kV, and source current of 100 µA, using a Cu 0.11 mm filter, with an exposure time of 6180 ms and a rotation step of 0.2° over 180°/360°. Reconstruction was performed in NRecon with beam-hardening correction set to 60%, ring-artifact correction set to 24, and Gaussian smoothing of 2. To distinguish titanium, calcium phosphate, and air, grayscale histograms were segmented in CTAn using global 0–65 (of 0–255) thresholds; the same threshold values were applied uniformly across all groups. Partial-volume voxels at phase boundaries were handled by <5 voxel, 3D despeckle operation and opening (3D) morphological operations, round Kernel, radius of 2, and metal-induced artifacts adjacent to titanium struts were not applicable. As-built strut diameter, open pore width, total porosity, strut surface area, tortuosity, and pore connectivity were quantified from pre-infiltration micro-CT scans of uncoated lattices in CTAn, using a fixed volume of interest (5 mm diameter × 4.6 mm height) applied identically to all specimens; values are reported for four independent specimens per configuration.

### 2.6. Statistical Analysis

This study was primarily designed as a materials characterization investigation, and the data presented are descriptive in nature. Due to the exploratory scope and limited sample size per condition, no inferential statistical analyses were performed. Future studies incorporating larger datasets and biological testing will include appropriate statistical comparisons.

## 3. Results

### 3.1. Fabrication and Macroscopic Appearance of Titanium Lattices

**Figure 3:**
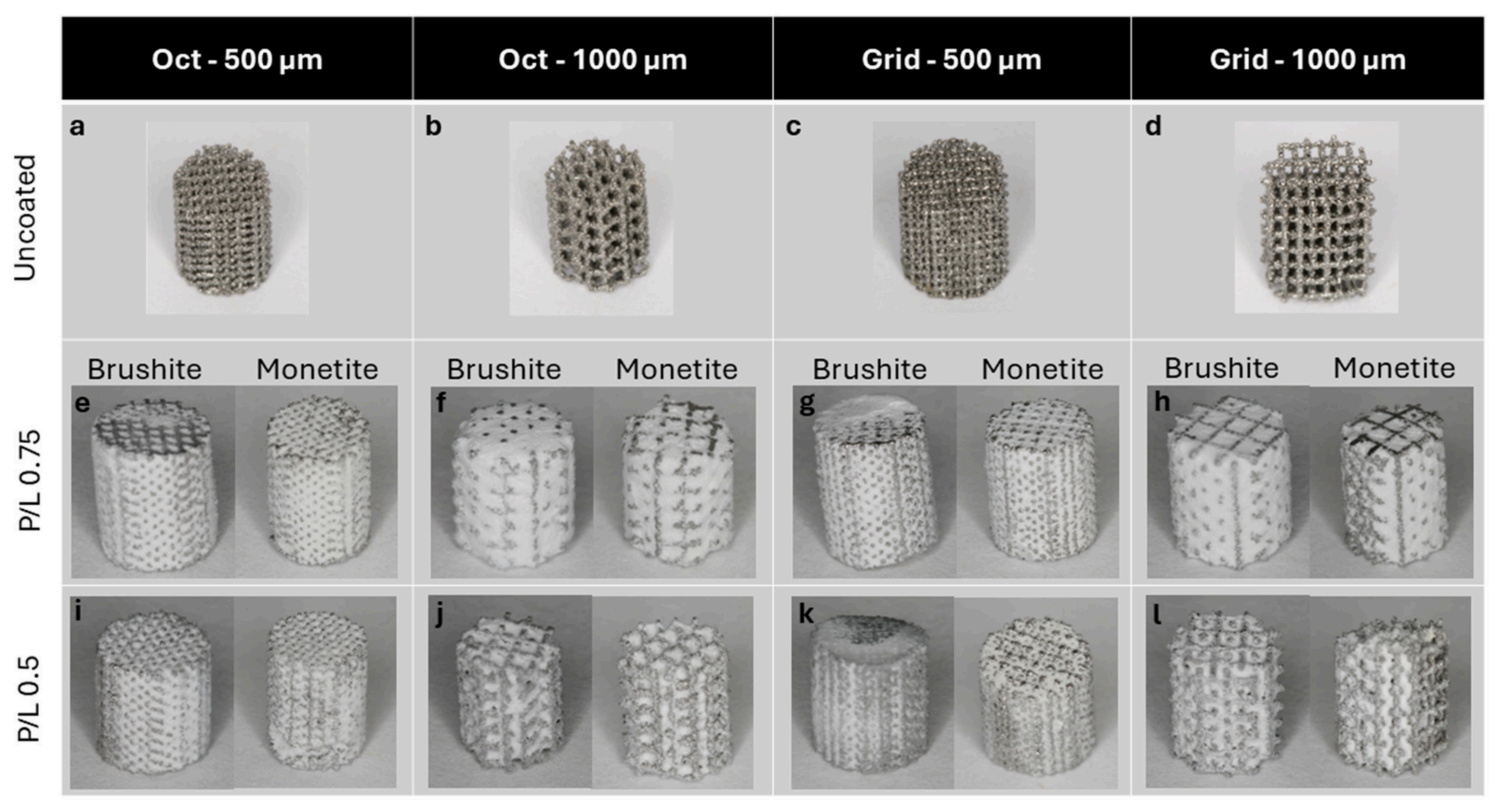
Digital photographs of uncoated, brushite-coated, and monetite-coated titanium lattices at varying geometries, strut spacings, and coating viscosities. (**a**–**d**) Uncoated lattices; (**a**) octagon lattice with strut spacing of 500 µm; (**b**) octagon lattice with strut spacing 1000 µm; (**c**) grid lattice with strut spacing 500 µm; (**d**) grid lattice with strut spacing 1000 µm. (**e**–**h**) Lattices coated with P/L 0.75, brushite (left) and monetite (right); (**e**) octagon lattices 500 µm; (**f**) octagon lattices 1000 µm; (**g**) grid lattices 500 µm; (**h**) grid lattices 1000 µm. (**i**–**l**) Photographs of lattices coated with P/L 0.5, brushite (left), and monetite (right); (**i**) octagon lattices 500 µm; (**j**) octagon lattices 1000 µm; (**k**) grid lattices 500 µm; (**l**) grid lattices 1000 µm.

Scanning electron microscopy (SEM) images of the Ti-6Al-4V (Ti64) lattices obtained before and after ultrasonication cleaning demonstrated a marked reduction in the number of loosely adherent spherical titanium powder particles on the lattice surfaces. Residual globular powder features that remained after sonication appeared fused to the lattice struts, indicating metallurgical bonding during the laser powder bed fusion (LPBF) printing process rather than loosely attached contaminants (Figure 2). Additively manufactured titanium lattices with both grid and octagonal architectures were successfully fabricated at nominal strut spacings of 500 µm and 1000 µm. All lattice configurations preserved their designed geometry following calcium phosphate (CaP) cement infiltration and subsequent drying or phase conversion treatments. No evidence of macroscopic deformation, fracture, or structural collapse was observed following coating procedures (Figure 3).

The ss-built architectural parameters, quantified from micro-computed tomography, are summarized in Table 2. Measured strut diameters (221–235 µm) exceeded the nominal design owing to partial particle fusion, and measured open pore widths (263 ± 12 µm and 280 ± 9 µm for the grid and octagon 500 µm designs; 776 ± 13 µm and 783 ± 9 µm for the 1000 µm designs) were correspondingly smaller than the nominal center-to-center strut spacings. Total porosity ranged from 54.8 ± 2.5% (grid, 500 µm) to 82.5 ± 3.7% (octagon, 1000 µm).

Visual inspection indicated that CaP cement application resulted in uniform surface coverage across the exposed titanium struts of the lattices. At the macroscopic level, brushite- and monetite-coated lattices exhibited similar overall appearance and surface coverage, with no visually distinguishable differences between the two calcium phosphate phases. In contrast, uncoated titanium lattices displayed the characteristic metallic surface finish and open porous architecture associated with LPBF-fabricated Ti64 structures. Representative photographs of uncoated lattices and lattices coated with brushite or monetite at both cement viscosities for all lattice configurations are shown in Figure 3.

### 3.2. Phase Identification of Calcium Phosphate by X-Ray Diffraction

**Figure 4:**
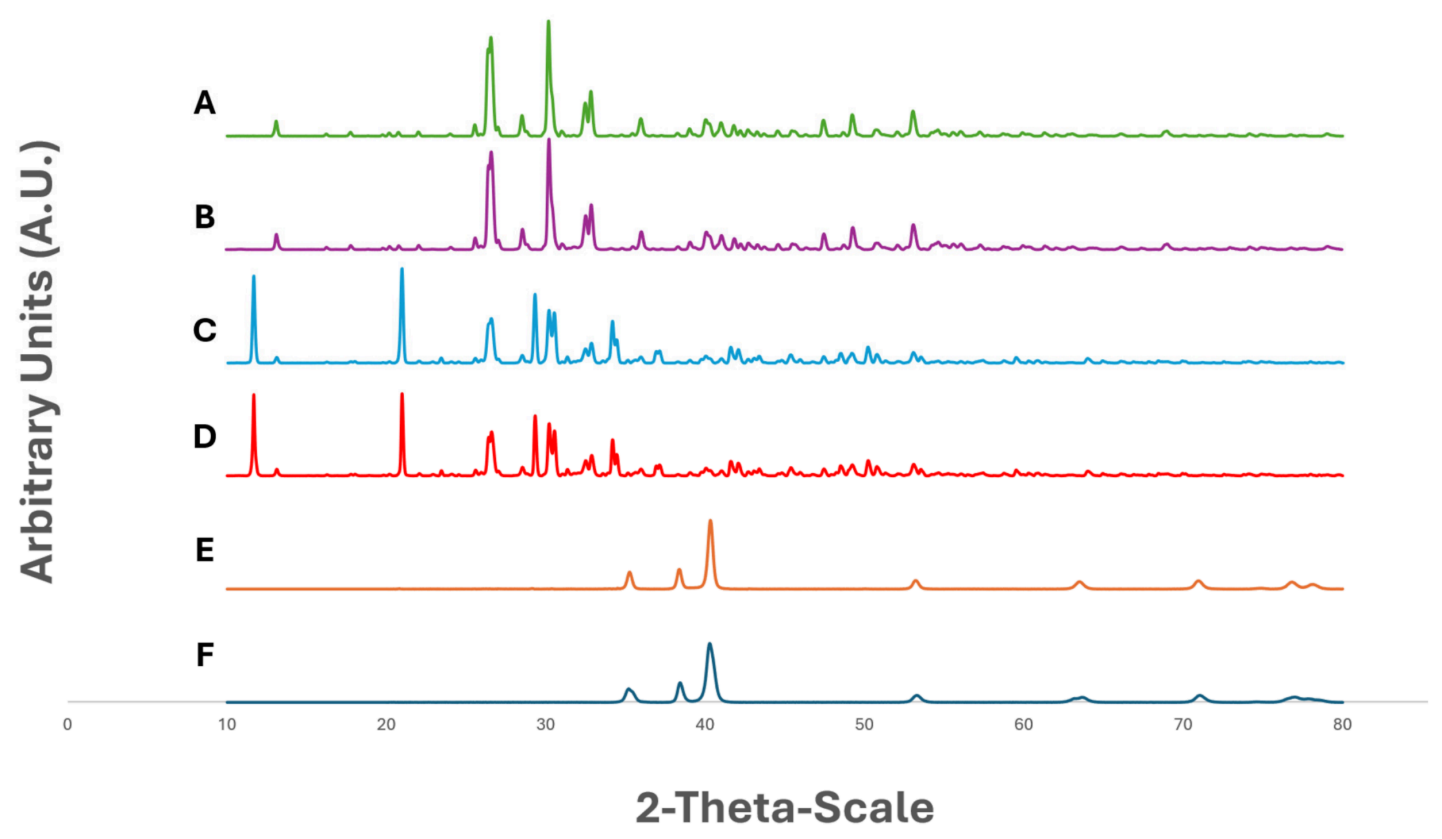
X-Ray Diffraction (XRD) analysis of Ti6Al4V and CaPs. (A–D) XRD was completed on brushite and monetite cements using the Bruker D8 Advance (A25), at a 2θ of 10° to 80° with a 0.02° step size, 1 s per step; (A) P/L 0.75, monetite; (B) P/L 0.5, monetite; (C) P/L 0.75 brushite; (D) P/L 0.5 brushite. XRD of titanium powder (F), and a Ti64 structure analogous to a lattice (E), was completed using the Bruker D8 Advance (A25) at a 2θ of 20° to 130° at a 0.02° step size and 1 s per step; (E) LPBF 3D-printed Ti64 structure analogous to the Ti64 lattices; (F) Ti64 powder prior to LPBF manipulation. The resulting diffraction pattern was compared in a database of known materials, confirming the titanium sample’s composition to be that of Ti6Al4V and the coating to be variations of calcium phosphate. (A–D) Peaks in the vicinity of 11° and 20° 2θ observed in Brushite, but not Monetite samples. (E,F) Ti64 structure and Ti64 powder show similar diffraction traces.

X-ray diffraction (XRD) analysis was performed on an uncoated Ti64 structure produced by laser powder bed fusion to verify the crystalline phase composition of the base material. The diffraction pattern of the uncoated structure exhibited peaks consistent with α-phase Ti-6Al-4V, with no detectable secondary phases or contamination, confirming the expected crystallographic structure of the printed titanium alloy (Figure 4). XRD analysis of the calcium phosphate coatings confirmed the presence of the intended dicalcium phosphate phases. Diffraction patterns obtained from brushite-coated lattices exhibited characteristic reflections corresponding to brushite (CaHPO_4_·2H_2_O). In contrast, samples subjected to autoclave treatment displayed diffraction patterns consistent with monetite (CaHPO_4_), confirming successful phase conversion. Notably, prominent reflections observed in the brushite patterns at approximately 2θ ≈ 11° and 20° were absent in the monetite patterns, consistent with the expected crystallographic differences between the hydrated and anhydrous dicalcium phosphate phases (Figure 4).

The diffraction peaks corresponding to brushite were indexed to characteristic crystallographic planes including (020), (021), and (041), consistent with patterns for CaHPO_4_·2H_2_O. Following thermal treatment, monetite peaks were identified and indexed to planes such as (120), (121), and (–202), confirming successful phase transformation to CaHPO_4_. These results confirm both successful deposition of brushite coatings and their subsequent conversion to monetite following the autoclave dehydration process.

The absence of prominent Ti-6Al-4V diffraction peaks in coated lattice samples is attributed to attenuation of the underlying metallic signal by the calcium phosphate coating layer, as well as the limited penetration depth associated with the Bragg–Brentano XRD configuration. As the coatings uniformly covered the lattice struts, the detected signal was dominated by the surface calcium phosphate phases rather than the underlying titanium substrate.

### 3.3. Surface and Microstructural Characterization by Scanning Electron Microscopy

**Figure 5:**
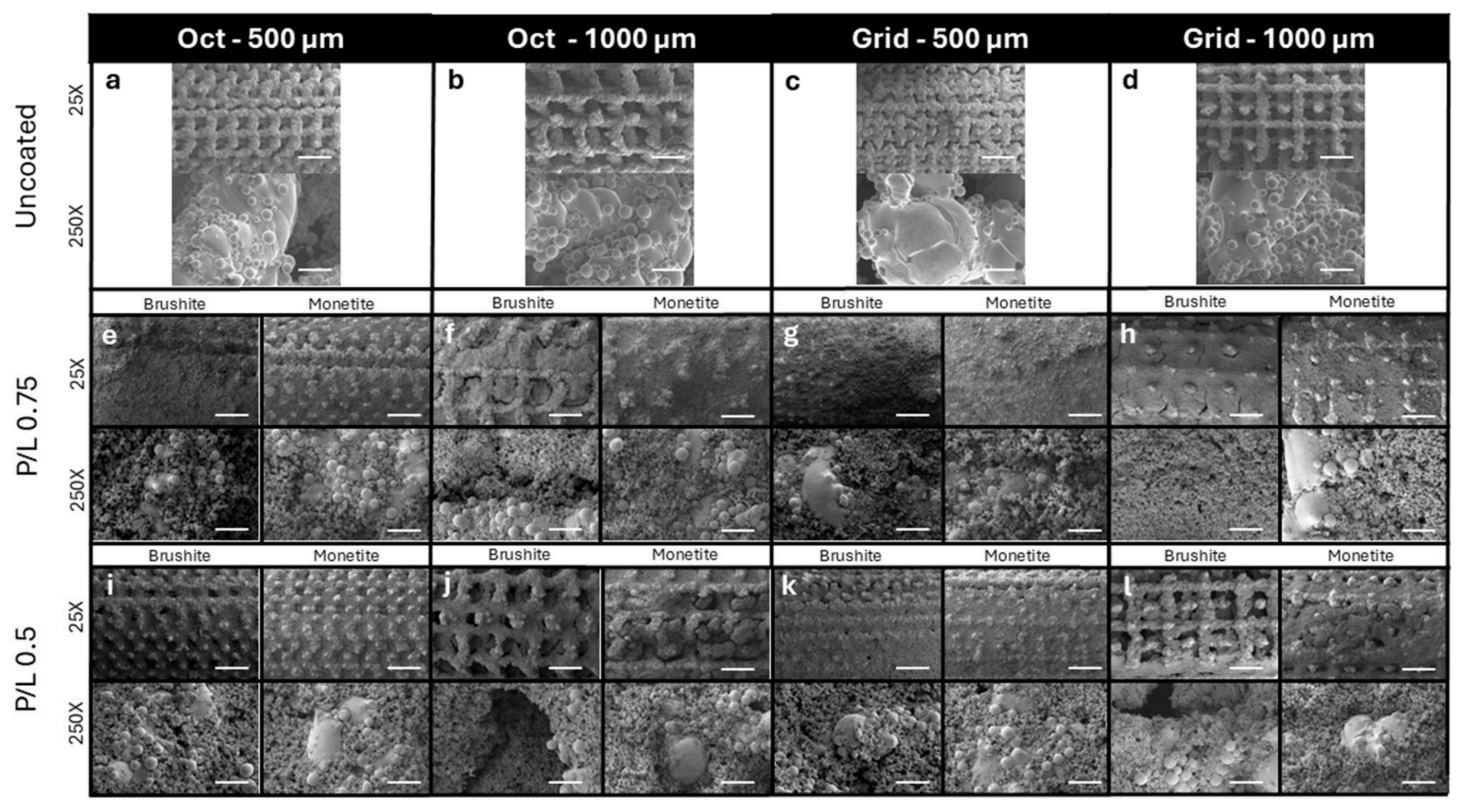
Scanning electron microscopy images of uncoated and coated lattices. (**a**–**d**) Uncoated lattices—25× magnification (Top), 250× magnification (Bottom); (**a**) octagon lattice with strut spacing 500 µm; (**b**) octagon lattice with strut spacing 1000 µm; (**c**) grid lattice with strut spacing 500 µm; (**d**) grid lattice with strut spacing 1000 µm. (**e**–**h**) Coated lattices: High viscosity, P/L 0.75; brushite (left), and Monetite (right) imaged at 25× magnification (Top), and 250× magnification (Bottom); (**e**) octagon lattices 500 µm; (**f**) octagon lattices 1000 µm; (**g**) grid lattices 500 µm; (**h**) grid lattices 1000 µm. (**i**–**l**) Coated lattices: Low viscosity, P/L 0.5; brushite (left) and monetite (right) imaged at 25× magnification (Top) and 250× magnification (Bottom); (**i**) octagon lattices 500 µm; (**j**) octagon lattices 1000 µm; (**k**) grid lattices 500 µm; (**l**) grid lattices 1000 µm. Scale bars on 25× magnification images represent 1000 µm. Scale bars on 250× magnification images represent 100 µm.

Scanning electron microscopy revealed clear morphological differences between uncoated titanium surfaces and calcium phosphate coated lattices. Uncoated titanium struts displayed the characteristic surface morphology associated with additively manufactured Ti64, including partially fused powder particles and microscale surface roughness arising from the layer-wise LPBF fabrication process (Figure 5). Following cement infiltration and setting, CaP-coated struts exhibited a continuous ceramic layer covering the underlying titanium surface. The coatings appeared to conform to the strut geometry, indicating successful deposition along both external and internal surfaces of the lattice structures (Figure 5).

Energy-dispersive spectroscopy (EDS) analysis confirmed the elemental composition of the coatings, with strong signals corresponding to calcium (Ca) and phosphorus (P), consistent with calcium phosphate deposition onto the titanium surfaces that showed their specific elemental signals based on their elemental composition (Ti, Al and V). While EDS confirmed the presence of calcium and phosphorus within the coatings, the measured Ca/P ratios did not consistently correspond to the theoretical stoichiometry of monetite (Ca/P = 1.0). This is attributed to the known limitations of EDS for quantitative compositional analysis in thin, heterogeneous coatings on metallic substrates, including electron beam interaction with the underlying Ti-6Al-4V lattice, surface roughness effects, and localized compositional variability within the calcium phosphate layer. As such, EDS results were interpreted as indicative of elemental presence rather than definitive phase composition. No extraneous elemental contaminants were detected within the sensitivity limits of the technique, supporting the chemical purity of the coatings (Supplementary Table S1).

### 3.4. Cement Infiltration and Internal Distribution Assessed by Micro-Computed Tomography

**Figure 6:**
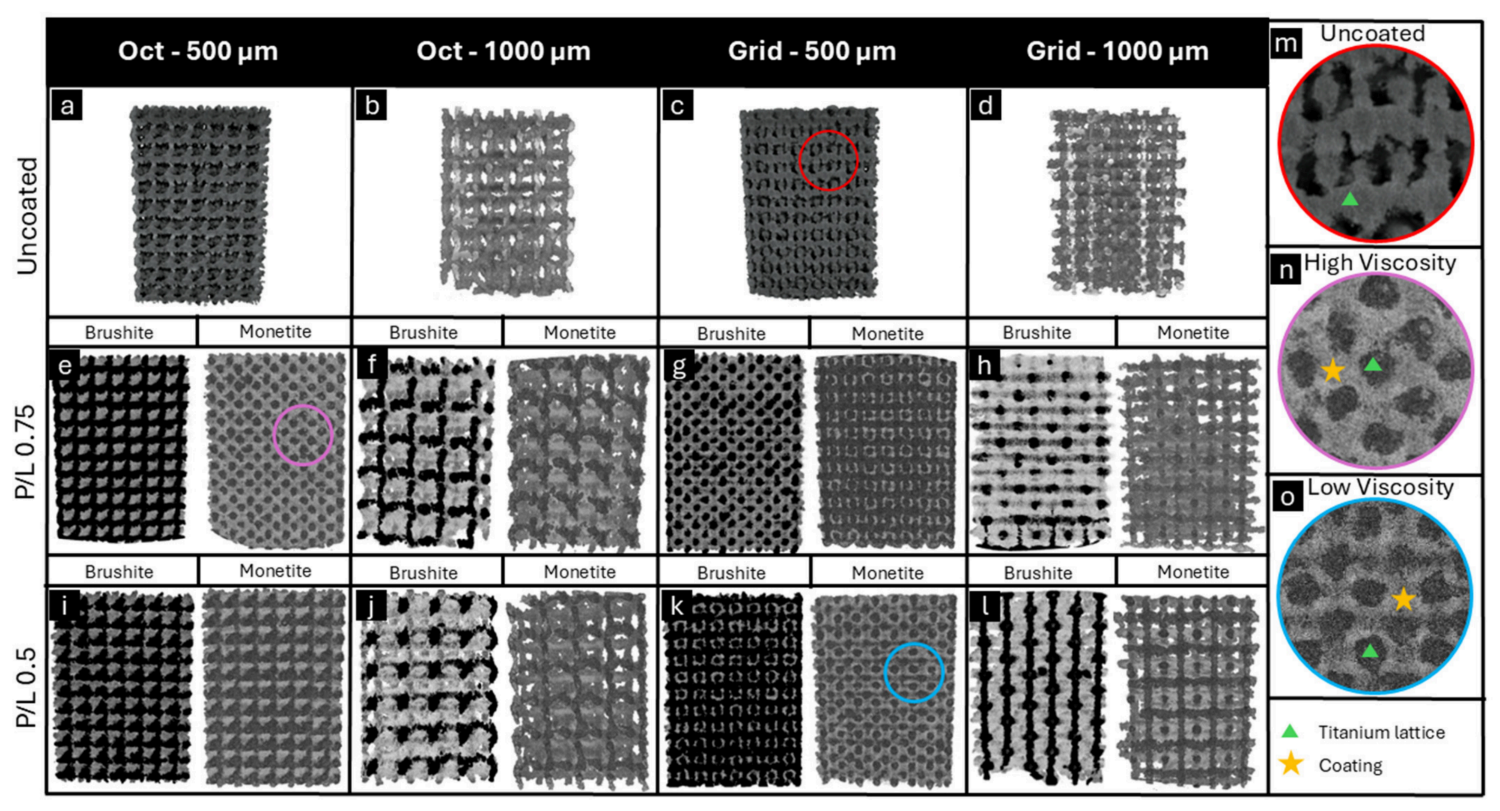
Micro-computed tomography analysis of uncoated and coated titanium lattices. Cross-sectional panels are orthogonal, longitudinal virtual slices taken through the center of each construct (internal sections), not external surface renderings. Micro-CT imaging (Bruker SkyScan 1272) was performed to evaluate coating infiltration and distribution within the internal lattice architecture, including coverage of internal struts. (**a**–**d**) Uncoated lattices: (**a**) Octagon lattice 500 µm; (**b**) octagon lattice 1000 µm; (**c**) grid lattice 500 µm; (**d**) grid lattice 1000 µm. (**e**–**h**) Coated lattices: P/L 0.75; brushite (Left) and monetite (Right); (**e**) octagon lattices 500 µm; (**f**) octagon lattices 1000 µm; (**g**) grid lattices 500 µm; (**h**) grid lattices 1000 µm. (**i**–**l**) Coated lattices: P/L 0.5; Brushite (Left); Monetite (Right); (**i**) octagon lattices 500 µm; (**j**) octagon lattices 1000 µm; (**k**) grid lattices 500 µm; (**l**) grid lattices 1000 µm. (**m**–**o**) Legend: Enlarged, representative lattice selections to assist with micro-CT image interpretation: (**m**) red circle, uncoated lattice selection; (**n**) purple circle, P/L 0.75 coated area; (**o**) blue circle, P/L 0.5 coated area; (**m**–**o**) Green triangle markers indicate darker-shaded visualization of titanium. Orange star markers indicate greyscale visualization of CaP coating.

Micro-computed tomography (micro-CT) imaging demonstrated extensive infiltration of calcium phosphate cement throughout all lattice configurations. Volumetric reconstructions revealed continuous distribution of the CaP phase from the external lattice surfaces to the central regions of the constructs, indicating effective penetration of the cement slurry into the porous architecture (Figure 6). Qualitative evaluation of scans showed cement penetration in both grid and octagonal designs at nominal strut spacings of 500 µm and 1000 µm. Cross-sectional micro-CT images confirmed the presence of CaP material along internal strut surfaces throughout the thickness of the constructs. Within the spatial resolution limits of the imaging modality, the cement distribution appeared relatively homogeneous, with no detectable non-infiltrated cores or architecture-dependent exclusion zones (Figure 6). Orthogonal, longitudinal cross-sectional slices taken through the center of each construct (Figure 6), that is, internal virtual sections rather than external surface views, showed calcium phosphate along internal strut surfaces through the full thickness, with no segmented non-infiltrated core resolved above the voxel-resolution limit. Differences among geometries and pore sizes were within the specimen-to-specimen variability are acknowledged and no inferential comparison was performed given the feasibility-scale sample size. Furthermore, no qualitative differences in penetration depth or internal coating distribution were observed between lattice geometries or strut spacings evaluated in this study. These findings indicate that the cement infiltration protocol employed was sufficient to achieve volumetric distribution of calcium phosphate within the additively manufactured titanium scaffolds. The as-built pore dimensions and porosity underlying these observations are reported in Table 2 (Section 3.1); the extensive infiltration described here was achieved across the full range of measured open pore widths (263–783 µm) and porosities (54.8–82.5%) represented by the four configurations.

## 4. Discussion

This study demonstrates that calcium phosphate cements can achieve extensive infiltration, without detectable geometry-dependent exclusion zones at the feasibility scale examined of laser powder bed fusion (LPBF) fabricated Ti-6Al-4V lattice scaffolds when appropriate processing conditions are applied. As this study was exploratory and descriptive in nature, quantitative statistical comparisons were not performed; however, consistent trends across all lattice configurations support the robustness of the observed infiltration behavior. Micro-computed tomography revealed continuous cement distribution from the external surfaces to the central regions of all lattice configurations evaluated, with no evidence of non-infiltrated cores or geometry-induced exclusion zones. This observation addresses an important but often implicit assumption in the design of hybrid metallic–ceramic scaffolds, namely that bioactive phases introduced at the scaffold surface will also access the internal pore network. While previous studies have reported the feasibility of calcium phosphate coatings on additively manufactured titanium scaffolds, most investigations have focused primarily on surface coatings or bulk mechanical performance rather than confirming penetration of the bioactive phase into the internal lattice architecture [30,31]. The present findings therefore provide direct volumetric evidence, rather than an assumption, that a bioactive phase introduced at the scaffold surface can be delivered uniformly to the internal pore network of an architected metal lattice. To our knowledge this internal distribution has more often been assumed than verified and confirming it across independent geometries and pore sizes is the principal contribution of this work.

The lattice geometries investigated in this study were designed with nominal strut spacings of 500 µm and 1000 µm, which are often compared to the pore-size range reported to support bone ingrowth. Numerous studies have suggested that pore sizes in the range of approximately 400–600 µm represent an optimal window for promoting osteogenesis, vascularization, and cell infiltration within porous biomaterial scaffolds [32,33,34,35]. These nominal values, however, describe center-to-center strut spacing rather than the open pore dimension available to cells and fluids; the as-built open pore widths measured here (Table 2) are considered in the following comparison. Because center-to-center strut spacing is not equivalent to open pore width, this comparison uses the measured as-built open pore widths (Table 2: 263 ± 12 µm for the grid and 280 ± 9 µm for the octagon 500 µm designs, and 776 ± 13 µm and 783 ± 9 µm for the respective 1000 µm designs) rather than nominal spacing. On this basis, the as-built 500 µm designs fall below the commonly cited ~400–600 µm osteogenic window, while the 1000 µm designs fall above it, representing a larger open architecture that may facilitate fluid transport and nutrient exchange but could influence mechanical stability and bone-bridging behavior.

However, with the coated and extensively cement-infiltrated lattices, the available pore space becomes negligible to non-existent for both 500 and 1000 µm. In vivo, this pore space would be expected to progressively increase as the calcium phosphate phase undergoes cellular-mediated resorption and is replaced by newly formed bone, as demonstrated in previous studies on brushite and monetite-based graft materials [26,30]. This remains to be studied in future investigations. Importantly, both architectures supported extensive cement infiltration under the conditions used in this study. These results suggest that the strut spacings investigated here are not only compatible with biological requirements for bone regeneration but are also permissive for the transport of calcium phosphate cement slurries into internal scaffold regions. From a design perspective, this finding highlights the feasibility of integrating bioactive calcium phosphate phases into architected titanium lattices without compromising pore sizes that are widely considered beneficial for bone regeneration.

Both grid and octagonal lattices exhibited volumetric calcium phosphate penetration at nominal strut spacings of 500 µm and 1000 µm, indicating that neither connectivity pattern nor characteristic feature size imposed a measurable transport barrier under the conditions evaluated. Consistent with this, the measured pore networks were highly interconnected across all configurations, with geometric tortuosity values between 1.20 and 1.39 (Table 2), indicating relatively direct transport paths in every architecture examined.

Lattice architecture is known to influence pore interconnectivity, tortuosity, and fluid transport pathways within architected scaffolds, factors that can strongly affect the movement of liquids or suspensions through porous structures [36,37]. Similarly, strut spacing and pore size are frequently cited as determinants of permeability and infiltration efficiency in porous biomaterials [38]. The absence of geometry-dependent exclusion zones observed here suggests that a range of clinically relevant lattice architectures can support uniform internal biofunctionalization when appropriate cement handling and infiltration strategies are employed. At the same time, these findings do not imply that lattice architecture is universally irrelevant to infiltration behavior; rather, they indicate that within the architectural space examined in this study, scaffold design parameters can be selected to optimize mechanical performance and biological function without necessarily compromising cement penetration.

The rheological behavior of dicalcium phosphate cements is strongly influenced by powder-to-liquid ratio, particle size distribution, and reaction kinetics. These parameters collectively affect viscosity, working time, and injectability, which in turn determine the ability of the cement to penetrate porous substrates. Previous studies have emphasized the importance of processing conditions in controlling the handling properties and biological performance of brushite- and monetite-based cements [21,23]. The present findings are consistent with cement workability being an important processing factor for internal infiltration within additively manufactured titanium lattices, although we did not perform rheological measurements and therefore describe the formulations by powder-to-liquid ratio rather than by measured viscosity. From a design perspective, this indicates that cement formulation should be considered a key processing variable alongside scaffold architecture when developing composite constructs that combine structural metallic frameworks with resorbable calcium phosphate phases.

Within the architectural space examined, lattice geometry and pore size did not appear to impose a detectable transport barrier at the feasibility scale studied; the processing variable we varied was cement workability, controlled through powder-to-liquid ratio. Both workable formulations (P/L 0.5 and P/L 0.75) were compatible with the infiltration procedure, whereas the highest-ratio formulation (P/L 1.0) was excluded prior to infiltration owing to inadequate workability. We therefore frame formulation as a practical processing prerequisite rather than as a quantified rheological determinant.

Two workable brushite cement formulations (P/L 0.5 and 0.75) consistently achieved extensive penetration across all lattice configurations, whereas the highest-ratio formulation (P/L 1.0) proved difficult to manipulate and was excluded from further evaluation prior to infiltration assessment due to inadequate workability. This observation highlights that infiltration behavior is associated with the coupled interaction between scaffold architecture and the flow characteristics of the cement slurry [39,40]. In highly interconnected porous networks such as those produced by additive manufacturing, cement workability may influence whether the slurry is able to traverse tortuous internal pathways before the onset of setting reactions [39,41].

From a mechanistic perspective, successful volumetric infiltration of the calcium phosphate cements can be understood as the result of favorable interactions between scaffold permeability and cement rheology. Architected titanium lattices produced by LPBF exhibit highly interconnected pore networks that enable capillary-driven flow and gravitational penetration of liquid suspensions when viscosity and yield stress remain within an appropriate range [39,41]. When the powder-to-liquid ratio produces a slurry with sufficiently low viscosity and adequate working time, the cement can traverse interconnected pores before the onset of significant setting reactions. In contrast, excessive viscosity increases resistance to flow and may prevent slurry transport into deeper lattice regions, particularly in tortuous pore pathways. P/L ratio was varied to identify a workable formulation that ensured full passage while limiting water content to avoid post-conversion porosity. The present results therefore suggest that achieving volumetric biofunctionalization requires alignment between scaffold permeability (controlled by lattice architecture and pore size) and cement rheological behavior. This coupled design principle provides a useful framework for future development of hybrid metallic–ceramic scaffolds in which structural architecture and biomaterial formulation are optimized simultaneously to achieve both mechanical integrity and uniform biological functionality.

X-ray diffraction analysis confirmed the expected crystalline phases of both the titanium substrate and the calcium phosphate coatings. Diffraction patterns of the uncoated structures were consistent with Ti-6Al-4V alloy, with characteristic reflections confirming the α-phase titanium structure produced during LPBF fabrication. Analysis of the calcium phosphate coatings verified the formation of brushite following cement setting and demonstrated successful conversion to monetite after thermal dehydration. This phase conversion was evidenced by the disappearance of characteristic brushite peaks at approximately 2θ ≈ 11° and 20° and the appearance of reflections associated with monetite. The presence of sharp diffraction peaks in both titanium and calcium phosphate phases further indicates the crystalline nature of the materials produced during the fabrication and coating processes.

The findings of this study are a materials-processing step toward, rather than a demonstration of, patient-specific implants for maxillary and craniofacial reconstruction. Large craniofacial defects resulting from oncologic resection, trauma, or congenital anomalies often require implants capable of providing immediate structural support while simultaneously supporting bone regeneration. The ability to achieve uniform volumetric infiltration of calcium phosphate within porous titanium scaffolds suggests a promising strategy for combining the mechanical reliability of metallic implants with the biological advantages of resorbable osteoconductive materials. Such hybrid constructs may be particularly advantageous in the maxilla, where bone density is relatively low, and regeneration must occur within a predominantly trabecular environment.

Despite these promising findings, the present study represents a feasibility-scale materials-processing study, and several limitations should be acknowledged. With limited sample size per condition, no inferential statistical analysis was performed, and quantitative metrics are reported descriptively. Micro-CT analysis was performed at a finite voxel resolution and was not cross-validated against destructive physical cross-sections. Attempts to saw or split the infiltrated lattices in half dislodged and destroyed the calcium phosphate coating at the cut face, precluding reliable region-resolved BSE-SEM/EDS on physical sections, so sub-resolution non-infiltrated regions cannot be fully excluded. In addition, we did not perform rheological measurements; formulations are described by powder-to-liquid ratio rather than by measured viscosity, yield stress, or working time. The coating–substrate adhesion was not quantified; because interfacial bond strength governs coating retention during handling, implantation, and loading, it is a prerequisite for translational use and is the primary objective of the next stage of this work, with planned pull-off tensile testing (ASTM C633-type), scratch testing, gravimetric coating-retention after controlled ultrasonication, and interfacial SEM. Mechanical performance of the hybrid constructs and biological responses (biocompatibility, vascularization, new-bone histology, and immune/macrophage response) were not evaluated. Finally, successful infiltration of 5 mm open cylindrical lattices under immersion with agitation does not establish uniform penetration into full-scale, geometrically complex, partially enclosed patient-specific implants; scale-up validation in clinically representative geometries is a defined next step.

Building on the present results, ongoing work will focus on evaluating the mechanical performance and degradation behavior of these hybrid constructs, as well as assessing cellular responses and bone regeneration in biologically relevant systems. Integration of patient-specific design workflows with additive manufacturing and controlled biomaterial functionalization may ultimately enable the development of customized implants capable of restoring both structural integrity and biological function in complex craniofacial defects. Taken together, the findings of this study demonstrate the feasibility of integrating additively manufactured titanium architectures with resorbable calcium phosphate phases to create hybrid constructs that combine structural reliability with volumetric bioactivity. By establishing preliminary processing conditions that enable extensive internal infiltration of dicalcium phosphate cements across multiple lattice architectures, this work provides a critical foundation for the development of next-generation craniofacial implants capable of supporting bone regeneration throughout their entire volume. The convergence of additive manufacturing, architected porosity, and bioactive ceramic functionalization offers a promising pathway toward patient-specific implants that more closely replicate the structural and biological characteristics of native bone.

From a clinical perspective, hybrid constructs of this type are of prospective interest for patient-specific craniofacial reconstruction, particularly for large maxillary defects requiring both structural support and biological regeneration. Realizing that potential will depend on first establishing coating adhesion, load-bearing performance, and biological response; the present work contributes only the infiltration and phase-conversion basis on which those subsequent evaluations can be built. This approach may reduce reliance on autologous grafting and associated donor-site morbidity, while enabling customized solutions tailored to complex anatomical defects. Continued investigation integrating mechanical characterization, degradation behavior, and biological performance will be essential to fully realize the translational potential of these hybrid constructs for craniofacial and orthopedic reconstruction.

## 5. Conclusions

This study demonstrates that dicalcium phosphate cements can achieve extensive volumetric infiltration within additively manufactured Ti-6Al-4V lattice scaffolds across multiple geometries and pore sizes when appropriate rheological conditions are applied. Micro-CT analysis showed penetration to the lattice core across both geometries and pore sizes, without detectable exclusion zones of calcium phosphate throughout the lattice structures, while SEM, EDS, and XRD verified coating continuity, composition, and phase conversion from brushite to monetite. Importantly, cement workability (P/L ratio) was an important practical parameter for successful infiltration, highlighting the need for coordinated design of scaffold architecture and biomaterial formulation. These findings establish the processing conditions required to achieve uniform, architecture-independent infiltration of resorbable calcium phosphate throughout LPBF titanium lattices, and confirm controlled conversion of the deposited phase from brushite to monetite. Establishing coating–substrate adhesion, hybrid mechanical performance, and biological response are defined next stages required before translational conclusions for craniofacial reconstruction can be drawn.

## Figures and Tables

**Table 1:** Workflow and preparation of sixteen unique coated lattice types *.

Lattice Shape	Octagon				Grid			
Strut Spacing	500 µm		1000 µm		500 µm		1000 µm	
P/L Ratio	0.5	0.75	0.5	0.75	0.5	0.75	0.5	0.75
Brushite Coated	Brushite	Brushite	Brushite	Brushite	Brushite	Brushite	Brushite	Brushite
	P/L 0.5	P/L 0.75	P/L 0.5	P/L 0.75	P/L 0.5	P/L 0.75	P/L 0.5	P/L 0.75
	Octagon 500 µm	Octagon 500 µm	Octagon 1000 µm	Octagon 1000 µm	Grid 500 µm	Grid 500 µm	Grid 1000 µm	Grid 1000 µm
Monetite Converted	Monetite	Monetite	Monetite	Monetite	Monetite	Monetite	Monetite	Monetite
	P/L 0.5	P/L 0.75	P/L 0.5	P/L 0.75	P/L 0.5	P/L 0.75	P/L 0.5	P/L 0.75
	Octagon 500 µm	Octagon 500 µm	Octagon 1000 µm	Octagon 1000 µm	Grid 500 µm	Grid 500 µm	Grid 1000 µm	Grid 1000 µm

* Four titanium lattice architectures categorized by geometry and strut spacing (octagon and grid geometries, each printed at 500 µm and 1000 µm strut spacing) were fabricated and coated with brushite cements prepared at P/L 0.5, P/L 0.75. Brushite-coated lattices underwent thermal dehydration (132° C, 45 min) to convert the brushite coating to monetite. This process resulted in sixteen unique lattice types classified by geometry and strut spacing, P/L coating ratio, and brushite or monetite coating type. Specimens belonging to each of the sixteen lattice types were labelled, weighed, and characterized for comparison.

**Table 2:** As-built architectural and microstructural characteristics of the laser powder bed fusion (LPBF) Ti-6Al-4V lattices determined by micro-computed tomography.

Parameter	Grid, 500 µm	Octagon, 500 µm	Grid, 1000 µm	Octagon, 1000 µm
Strut diameter (µm)	233.5 ± 12.0	221.2 ± 9.1	234.8 ± 11.3	222.8 ± 11.2
Open pore width (µm)	263.2 ± 12.4	279.5 ± 7.1	775.5 ± 12.7	783.2 ± 8.8
Total porosity (%)	54.8 ± 2.5	61.8 ± 1.4	81.8 ± 3.7	82.5 ± 3.7
Strut surface area (mm^2^)	1596.2 ± 11.9	1537.5 ± 23.2	886.2 ± 8.2	881.5 ± 16.9
Tortuosity	1.20 ± 0.10	1.39 ± 0.08	1.22 ± 0.07	1.31 ± 0.06
Pore connectivity	Highly interconnected	Highly interconnected	Highly interconnected	Highly interconnected

Values are presented as mean ± standard deviation (*n* = 4 independent specimens per configuration). Strut diameter and open pore width are as-built dimensions and differ from the nominal (CAD) center-to-center strut spacings of 500 and 1000 µm, reflecting partial particle fusion inherent to the LPBF process; open pore width is reported as the measured open dimension rather than the nominal strut spacing. Total porosity was derived from the segmented titanium volume fraction within a fixed volume of interest (5 mm diameter × 4.6 mm height) applied identically to all specimens. Strut surface area denotes the total object (strut) surface within the volume of interest. Tortuosity describes the mean geometric tortuosity of the pore network (1.0 = a perfectly straight path). Pore connectivity was qualitatively assessed as a single, fully interconnected pore network in all configurations.

## Data Availability

The original contributions presented in this study are included in the article/Supplementary Materials. Further inquiries can be directed to the corresponding author.

## Supplementary Materials

The following supporting information can be downloaded at: https://www.mdpi.com/article/10.3390/ma19183973/s1, Table S1. Summary of EDS data.

## Abbreviations

The following abbreviations are used in this manuscript:

β-TCP	Beta-tricalcium phosphate
CaPs	Calcium Phosphates
DCPA	Dicalcium phosphate anhydrous (Monetite)
DCPD	Dicalcium phosphate dihydrate (Brushite)
DCP(s)	Dicalcium phosphate(s)
HA	Hydroxyapatite
LPBF	Laser powder bed fusion
MCPM	Monocalcium phosphate monohydrate
Micro-CT	Micro-computed tomography
P/L	Powder-to-liquid ratio
SEM	Scanning Electron Microscopy
Ti64	Titanium alloy Ti6Al4V
XRD	X-Ray Diffraction
3D	Three-dimensional
