# Supplementary material for: Volumetric Infiltration of Brushite and Monetite Cements into Laser Powder Bed Fusion Ti-6Al-4V Lattices: A Feasibility Study of Lattice Geometry and Cement Formulation
Source: Materials (Basel). 2026 Sep 18;19(18):3973. doi: 10.3390/ma19183973 (PMC13608174; doi:10.3390/ma19183973)
Supplement: Supplementary file 1 [file materials-19-03973-s001.zip › materials-4529793-supplementary.pdf]

Supplementary Table S1. Summary of EDS data.

Material	Ca (weight %)	P (weight %)	O (weight %)	Ti (weight %)	Al (weight %)	V(weight %)
Uncoated Ti64 Lattice	4.6 ± 0.0	N/A	N/A	76.6 ± 0.3	4.1 ± 0.0	3.9 ± 0.0
Brushite Cement	34.6 ± 0.1	22.5 ± 0.2	42.9 ± 0.2	N/A	N/A	N/A
Monetite Cement	30.4 ± 0.1	19.2 ± 0.1	50.4 ± 0.2	N/A	N/A	N/A
Brushite Coated Lattice	27.5 ± 0.1	16.3 ± 0.1	36.7 ± 0.2	17.8 ± 0.1	0.4 ± 0.0	1.1 ± 0.1
Monetite Coated Lattice	23.3 ± 0.1	7.6 ± 0.1	30.8 ± 0.5	25.1 ± 0.2	0.7 ± 0.0	0.2 ± 0.2

Data presented as mean weight percent values with weight percent errors (n = 5).

Ca – elemental calcium

P – elemental phosphorus

O – elemental oxygen

Ti – elemental titanium

Al – elemental aluminum

V – elemental vanadium
